# A Retrospective Glance

**Published:** 1886-06

**Authors:** Herbert A. Birdsall


### A RETROSPECTIVE GLANCE.

BY HERBERT A. BIRDSALL, D. D. S.

Read Before the Eighth District Dental Society of the State of New York.

Beginning the study of statesmanship, or of political economy, the student is first directed to search history, and there trace out the formation and growth of civil institutions, from the simple associations of a primitive community through the various grades of their evolution unto the complex forms which present themselves to our view in modern society. May not a professional man in the same way strengthen his hold upon his position by finding how he came by it? To attain to a true understanding of a subject, perhaps there is no better place to begin than by turning back to find its beginnings, and also to find by what processes of evolution it has been developed.

It has been my good fortune to have access to a typical dental library of fifty years ago. It is the purpose of this paper to show, or perhaps rather to suggest, the relation of the dentistry of fifty years ago with the dentistry of to-day.

But to digress a moment. When the learned and erudite Knickerbocker wished to give us an account of the early history of Manhattan Island, in order that there might be no mistake in the premises he began with chaos, got together material for a world, which was suitably placed in its orbit; after which, by having man properly created, multiplied and distributed, he was able to come by easy and graceful steps to the telling of his story, sure of the ground behind him. This remark is thrown in as a sufficient precedent and excuse for turning back to glance over the longer tract of the more distant past.

"In the beginning," then—to use a well authorized expression—when the arts and sciences were entering the race, dentistry seems to have started early, and to have started well. The mummies of the ancient Egyptians have brought forth teeth filled with gold,* and specimens of bridge-work—which seems to be about the oldest form of dentistry—have been found both in Egyptian and Etruscan tombs. It is probable, also, that the Phœnicians, Arabs and Greeks had made some progress in the art, if not in the science of dentistry. But medicine and surgery in all their branches, having early deviated from their true course, were soon given over to alchemy, necromancy and magic. Men sought not after knowledge where it was to be found, but sat gazing into the smoke that perchance they might discern a form, or experimented with all manner of devices in search of a panacea. Dentistry fell out with the rest, and what little had been known was lost. Teeth were no longer considered in the light of organs to be rescued from destruction, but as amulets for the warding off of evil, or under varying circumstances, as omens of good or bad.

Through mediæval history the figure of darkness so frequently applied to affairs of the time in their social and political aspect, seems from the dental standpoint still more appropriately to apply. Dental defects and deformities are mentioned in tones of pity because they are considered without remedy. The familiar proverb which had its origin at that time, that a bad tooth was considered of all things the most desirable to be rid of, lets us into the secret. It was not until within the past hundred years that any form has been discerned in the smoke.

Having returned, then, to our subject, let us see what it contains. Let us first take the matter of theories concerning the causes of caries. Up to the time we have chosen to discuss,

---

* This remains to be proved. We hope that Dr. Van Marter, of Rome, who has so exhaustively studied Etrurian dentistry will soon perform the same office for Egypt.—EDITOR.

the two theories that divided the adherents were those of Fox and Bell. Briefly stated, they are as follows: From some cause, not very clearly made out, inflammation is excited in the membrane lining the cavity of a tooth (cavity with them means pulp-chamber), and in such inflammation separates from the bone with which it is in contact. Death of that part of the tooth speedily follows, and caries is the result. The Bell theory, like the other, rests on inflammation; but here it is not in the internal membrane, but in the tooth-bone itself. A blow, or the pressure of the teeth themselves against each other at their approximal surfaces, causes inflammation of the tooth-bone beneath the enamel, and gangrene and caries follow. It will be seen that the theories are alike, in that they both depend on inflammation, and both make caries to originate from within, making its way to the exterior. But at about our focal date there is a revival of investigation and discussion on this subject, and Robertson appears with a new theory. It was this: That decay was a purely chemical process, brought about by the decomposition of food and the presence of acid between and around the teeth. But to fortify himself he thought it necessary to hold the position that the teeth were extraneous and essentially unorganized bodies, and were in no sense to be considered as vital organs. This last theory seems to have been the generally accepted one at the time. Dr. Spooner, who was a very intelligent writer, says this: "There are two kinds of decay in teeth, which may be designated as internal and external. The first appearance of internal is marked by a peculiar dark-bluish spot which shines through the transparent enamel, the integrity of which appears to be perfect, while the first appearance of external caries is marked by erosion of the surface of the enamel."

As regards remedies, let us turn again to our library. Dr. E. Parmley says that "the benefits to be derived from plugging the teeth are so important that it is impossible to recommend it too strongly upon the public." In Dr. Spooner's book, published in 1836, just fifty years ago, I find the following. After hedging on all sides against quibble or contradiction, he lets slip this assertion: "The utility of plugging teeth for the cure of caries, as practiced by scientific dentists, is now known and appreciated by many persons; by more it is not sufficiently known or understood, while others distrust its utility altogether." Then follow several pages of facts and arguments to prove that teeth can be plugged to the advantage of the patient.

Of materials for stopping teeth let me glean a little from Kœcker. "Lead, tin and silver," he says, "are used for the purpose, but on account of the fact that they soon corrode he finds them more or less objectionable. Platina is more suitable than the above mentioned, but in consequence of the necessity of amalgamating some other metal with it to make it malleable, it is by this process rendered insufficient for the purpose." He finds it objectionable also because of the discolored appearance it gives the tooth, which is apt to mislead the dentist in the future into the idea that the tooth is still under the influence of decay.

"A composition," he continues, "is used by some in this country, and generally in France. It is composed of bismuth, tin and lead. It melts at a heat of boiling water, and is called fusible metal."

The next few paragraphs are taken up with showing the objections to this preparation. Let us look at some of them. They are interesting. After making the ordinary objections of corrosiveness, etc., he says that "the using of the metal at such a high degree of temperature destroys the vitality of the tooth;" and, as though that were not enough, he adds another, to the effect that "not only is the vitality of the tooth destroyed, but frequently also the whole surface of the healthy bone with which the tooth comes in contact;" an objection, it seems to us, which might well cause a moment's hesitation.

On the subject of cement filling materials Dr. Spooner, in his rather flowery language, has this to say:

"Various substances in the form of cements have been used in past ages, and are still, for the purpose of filling decayed and hollow teeth. All such substances are of very little use, and are avoided by honorable and well-informed dentists. Cements are composed of earthy substances which have the property of hardening under water, mixed with filings of metals, usually of zinc, in order to make them harder. Also of metals amalgamated with mercury. Any person at all acquainted with the principles on which a hollow tooth must be plugged, that it may be cured, will at once perceive that no cement can ever be devised that shall prove of much utility for the purpose. In order that carious teeth may be cured it is necessary that the decay be removed and the cavity be plugged airand water-tight. Cements do not, nor can they ever be devised to, effect this object." Rather a dagerous dip into the future.

Filing the teeth seems to have comprised a large part of the operative dentistry of the time, and occupies much space in their discussions.

On the subject of Pyorrhœa Alveolaris I find one or two short chapters, entitled "Scurvy of the Gums," which bear but slight resemblance to a modern discussion of the subject.

And last, but not most unimportant, I turn for information upon the subject which demands so much of our attention, and which fills so large a place in our discussions: Alveolo Dental Abscess. About all I can find upon the subject is in one little chapter in the back part of Dr. Spooner's book headed "Gum Biles." Spelled as pronounced, b-i-l-e-s. On reading it I find that sometimes they are not "Gum Biles" at all but that they frequently present themselves upon the cheek and neck, making offensive ulcers, or healed, unsightly scars. The only remedy is extraction.

Truly, from the smoke into which our fathers gazed so intently, yet saw but dimly, or not at all, the form, has appeared the full form of a genius who has done wonders, beyond the dreams of the magicians.

